# Inflammatory Microenvironment and Adipogenic Differentiation in Obesity: The Inhibitory Effect of Theobromine in a Model of Human Obesity In Vitro

**DOI:** 10.1155/2019/1515621

**Published:** 2019-01-20

**Authors:** Maria Pia Fuggetta, Manuela Zonfrillo, Cristina Villivà, Enzo Bonmassar, Giampiero Ravagnan

**Affiliations:** ^1^Institute of Translational Pharmacology CNR, Via Fosso del Cavaliere, 00133 Rome, Italy; ^2^Department of Systems Medicine, School of Medicine, University of Rome Tor Vergata, Rome, Italy

## Abstract

**Objective:**

Obesity is considered a clinic condition characterized by a state of chronic low-grade inflammation. The role of macrophages and adipocytokines in adipose tissue inflammation is in growing investigation. The physiopathological mechanisms involved in inflammatory state in obesity are not fully understood though the adipocytokines seem to characterize the biochemical link between obesity and inflammation. The aim of this work is to analyze the effect of theobromine, a methylxanthine present in the cocoa, on adipogenesis and on proinflammatory cytokines evaluated in a model of fat tissue inflammation in vitro.

**Methods:**

In order to mimic in vitro this inflammatory condition, we investigated the interactions between human-like macrophages U937 and human adipocyte cell lines SGBS. The effect of theobromine on in vitro cell growth, cell cycle, adipogenesis, and cytokines release in the supernatants has been evaluated.

**Results:**

Theobromine significantly inhibits the differentiation of preadipocytes in mature adipocytes and reduces the levels of proinflammatory cytokines as MCP-1 and IL-1*β* in the supernatants obtained by the mature adipocytes and macrophages interaction.

**Conclusion:**

Theobromine reduces adipogenesis and proinflammatory cytokines; these data suggest its potential therapeutic effect for treating obesity by control of macrophages infiltration in adipose tissue and inflammation.

## 1. Introduction

Obesity has been recognized as a disease associated with an increased risk of cardiovascular disorders, type 2 diabetes mellitus (T2DM), certain types of cancer, osteoarthritis, and asthma [1]. Obesity is characterized by an excessive accumulation of white adipose tissue (WAT) that can result from both hyperplasia and hypertrophy of adipocytes. Adipogenesis is a complex process involving the conversion of undifferentiated preadipocytes into differentiated adipocytes accompanied by changes in cell morphology, gene expression, and susceptibility to various hormonal influences [2, 3]. Obesity is characterized by a chronic state of low-grade inflammation with progressive immunocompetent cell infiltration into adipose tissue. It is now accepted that WAT is the primary source of many proinflammatory cytokines and adipokines [4]. In the adipose tissue, excessive infiltration of macrophages is crucial for the development of an inflammatory state [5]. The crosstalk between macrophages and adipocytes maintains a vicious cycle that sustains adipose tissue inflammation [6]. Inflammatory cytokines such as IL-1*β* and TNF*α*, mainly produced by macrophages, free fatty acids, and abnormal adipokine secretions by adipocytes are involved in this loop [7–9]. In turn, IL-1*β* directly stimulates the production and secretion of cytokines and chemokines like IL-6 and MCP-1 by human preadipocytes [10]. Furthermore, MCP-1 facilitates infiltration of macrophages into WAT [11, 12]. The secretion of these, and other inflammation-related factors, is considered to support the development of the major obesity-associated diseases and the metabolic syndrome. In fact, these proinflammatory molecules can block insulin function in adipocytes via autocrine/paracrine signaling causing systemic insulin resistance [13–15].

The control of adipogenesis, with numeric decrease of adipocytes, the reduction of lipid content of adipocytes, and the control of cytokine production that modulate inflammatory responses would be an effective strategy to prevent and treat obesity.

Recent studies reported that various phytochemicals show antiobesity effects, including inhibition of adipocyte differentiation and adipogenesis [16].

Cocoa is a rich source of antioxidants due to its content in flavanols [17] so the consumption of cocoa has been associated with the prevention or attenuation of several health disorders, including hypertension, endothelial dysfunction, inflammation, and oxidative stress [18]. Much less is known about the effects of cocoa on obesity [19].

Theobromine (TB, 3,7-dimethylxanthine), a caffeine derivative mainly found in cocoa beans and dark chocolate, belongs to methylxanthines, a class of alkaloids of plant origin that include caffeine and theophylline [20]. Scarce information is presently available on the biological activity of TB on human WAT. It is well known that TB is able to inhibit adipogenic differentiation of primary adipose-derived 3T3-L1 mouse cells through the suppression of adipogenesis-related factors [21, 22]. In particular, the antiadipogenic effects of TB on human preadipocytes as well as inflammatory cytokines release by infiltrated macrophages has not yet been examined. In the present investigation, we used a Simpson-Golabi-Behmel syndrome (SGBS) preadipocyte cell strain [23, 24] that is widely considered to be a representative in vitro model of human preadipocytes. Using these preadipocytes, we studied the suppressive activity of TB on cell cycle, adipogenic differentiation, and on the production of cytokine in a human model of adipocyte/macrophage interaction in vitro.

The preliminary results show that TB reduces the human adipogenesis in vitro significantly, and it is able to control the inflammatory cytokines release suggesting that this natural constituent of cocoa could be capable, at least in part, to counteract essential steps leading obesity.

## 2. Materials and Methods

### 2.1. Reagents and Chemicals

Cell culture media as RPMI 1640 GlutaMAX™ was purchased from Gibco (CA, USA), DMEM/Ham's F12 from Biowest SAS (France), and fetal bovine serum (FBS) from Corning Life Sciences. Penicillin and streptomycin were purchased from Biowest (SAS France).

Theobromine, biotin, pantothenate, human transferrin, insulin, hydrocortisone, triiodothyronine, dexamethasone, isobutyl-3-methylxanthine (IBMX), rosiglitazone, Oil Red O, phorbol-12-myristate-13-acetate (PMA), bromuro di 3-(4,5-dimetiltiazol-2-il)-2,5-difeniltetrazolio (MTT), and isopropanol were purchased from Sigma-Aldrich Chemical (St. Louis, MO).

Theobromine was diluted in heated RPMI 1640 containing NaOH (10 mM). The same solution was used as vehicle control.

### 2.2. Cell Culture: Promonocytic and Adipocyte Differentiation

Human promonocytic cell line U937 cells (ATCC) were cultured in RPMI 1640 GlutaMAX™ medium containing 10% FCS, 100 U/ml penicillin, and 100 *μ*g/ml streptomycin hereafter called complete medium (CM). The cells were maintained at 37°C in a 5% CO2 humidified incubator.

U937 cells (2 × 10^6^/ml) were differentiated in vitro into macrophages (dU937) by induction with PMA (10 ng/ml) in a CM. Macrophage-conditioned medium (hereafter referred as MacCM) from dU937 was collected after additional 48 h of incubation in a RPMI 1640 GlutaMAX serum-free basal medium and cleared by centrifugation.

Cells were cultured at 37°C in a 5% CO2 humidified incubator.

Human preadipocytes, SGBS cells [23], were kindly supplied by Dr. M. Wabitsch (Department of Pediatrics and Adolescent Medicine, Ulm University Medical Center, Ulm, Germany).

Briefly, adipogenic differentiation of SGBS cells was induced in a serum-free DMEM/F12 medium supplemented with 10 *μ*g/ml transferrin, 10 nM insulin, 200 pM thyroid hormone, and 0.1 *μ*M cortisol. For the first 4 days, 2 *μ*M rosiglitazone, 250 *μ*M isobutylmethylxanthine (IBMX), and 25 nM dexamethasone were added. At the end of these four days, the medium was replaced by a differentiation medium (DM) excluding dexamethasone, IBMX, and rosiglitazone. DM was changed every day for four days.

SGBS cultures were used for experiments when the differentiation rate was ≥85%.

In order to evaluate adipocyte differentiation, Oil Red O staining was performed on day 14 to detect the accumulated lipid droplets in dSGBS cells. On day 14 of differentiation, dSGBS cells were washed with phosphate-buffered saline (PBS), fixed with 4% formaldehyde for 15 min at room temperature, and then rinsed with 60% isopropanol.

The differentiated adipocytes were then stained with Oil Red O solution at room temperature for 20 min. After removing the staining solution, the cells were washed at least 1 time with 60% isopropanol and 3 times with distilled water and dried. The stained lipid droplets were visualized by light microscopy.

In addition, to obtain a quantitative evaluation of adipocyte differentiation, stained lipid droplets were solubilized in 100% isopropanol and measured by determining the optical density (OD) at 490 nm using a Multiskan EX, Labsystems analyzer.

Morphologically differentiated adipocytes (dSGBS cells) were used for experiments 8 days after the initiation of adipogenic differentiation.

### 2.3. Experimental Protocols and Theobromine (TB) Treatment

In order to evaluate the effect of TB on cell viability, SGBS and U937 cells, untreated or treated with PMA alone (10 ng/ml) or in combination with LPS (1 *μ*g/ml), were plated into 96-well plates (BD Falcon) and allowed to grow at 37°C in a 5% CO2 for 24 hours. Thereafter, the cultures were exposed to graded concentrations of TB (i.e., from 25 to 200 *μ*g/ml) for additional 48 hours and tested in a MTT assay. Briefly, 20 *μ*l of MTT (5 mg/ml) was added to each well and incubated for 4 hours; then, 100 *μ*l of solubilization solution was added. The formazan production was detected by determining the optical density (OD) at 570 nm using a Multiskan EX, Labsystems analyzer.

To evaluate the effect of TB on adipocyte differentiation, preadipocytes SGBS cells were seeded in 96-well plates (Falcon). On day 8, after induction of adipocyte differentiation in DM, cells were treated with TB (100 *μ*g/ml); control DM and DM-containing TB were changed every two days. At the end of adipogenic differentiation, on day 14, the Oil Red O staining was performed to display the accumulated lipid droplets in differentiated adipocytes (i.e., dSGBS). To quantify staining of fat droplets, Oil Red stain was extracted by adding isopropanol to each well. The aliquots of 100 *μ*l were transferred to 96-well plates and immediately read in an Elisa reader at 490 nm.

We have performed three experimental protocols in order to assess the effect of TB on the interaction between adipocyte and macrophage: directly (contact cell to cell) or indirectly through the supernatant of differentiated macrophages dU937 (i.e., MacCM) and the supernatant of dSGBS cells (i.e., ApoCM) collected on day 9 of differentiation.

The first protocol was performed as follows: dSGBS cells at day 8 of differentiation were incubated in a 24-well plate (10^4^ cells/well), in MacCM, with or without TB (100 *μ*g/ml) for 48 h. At the end of TB treatment, conditioned medium was collected and used to evaluate the presence of IL-6, IL-1*β*, and MCP-1 by specific ELISA test.

The second protocol was performed as follows: dU937 cells were incubated in a 24-well plate (10^4^ cells/well, Falcon), in ApoCM, with or without TB (100 *μ*g/ml) for 48 h. At the end, the conditioned medium was collected and used to evaluate the presence of IL-1beta by specific ELISA test.

The third protocol was performed as follows: coculture experiments were performed cultivating dSGBS adipocytes with dU937 macrophages. In vitro differentiated dU937 macrophages were trypsinized and added to cultures of dSGBS adipocytes (on day 8 of adipogenic differentiation); the dSGBS/dU937 cell ratio was kept at 10 : 1 (i.e., 10^4^/10^3^ cell per well). The coculture of dSGBS/dU937 was performed in MacCM pooled from 3 independently macrophage culture. Treatment with TB (100 *μ*g/ml) was performed at the start of dSGBS/dU937 coculture and maintained for 48 h. The coculture supernatant was collected and centrifuged to remove debris and used to evaluate the presence of IL-6, IL-1*β*, and MCP-1 by specific ELISA.

### 2.4. Flow Cytometry and Analysis

The cell cycle distribution was analyzed by flow cytometry in preadipocytes SGBS untreated or treated with TB (at the concentration of 100, 50, and 25 *μ*g/ml) for 24, 48, and 72 h, and in differentiated dSGBS cells. SGBS and dSGBS cells were fixed overnight with 70% ethanol at 4°C and then were incubated with 10 *μ*g/ml of RNase A and 50 *μ*g/ml of propidium iodide at room temperature in the dark for 1 hour. Samples were acquired on FacsCalibur (BD Becton Dickinson) at 488 nm. The acquired FACS data were analyzed by ModFit LT software (Verity Software House, Inc.) to determine the percentage of cells in sub G1, G1, S, and G2/M phases.

### 2.5. Enzyme-Linked Immunosorbent Assay (ELISA)

The secretion of IL-6, IL-1*β*, and MCP-1 from dU937 and dSGBS cells alone or each other cocultived, treated, or not with TB (100 *μ*g/ml) was measured in the supernatants of cell cultures (see detailed protocols). To determine the IL-6 and IL-1*β* concentration in the supernatants, the commercially available ELISA kits (R&D Systems) were used according to the manufacturer's protocols. The MCP-1 protein levels were determined using the Human CCL2 (MCP-1) ELISA kit (Sigma-Aldrich, St. Louis, MO) following the manufacturer's instructions. The concentration of IL-6, IL-1*β*, and MCP-1 in the media was measured with an Elisa reader (Multiskan EX, Labsystems) at 450 nm.

### 2.6. Statistics

Data are represented as mean ± SEM of three independent experiments. Statistical significance was calculated by using one-way analysis of variants (ANOVA) and Dunnett or Tukey correction test, where *p* < 0.05 was considered as statistically significant.

## 3. Results and Discussion

### 3.1. Cell Cycle in Differentiated Adipocytes

**Figure 1:**
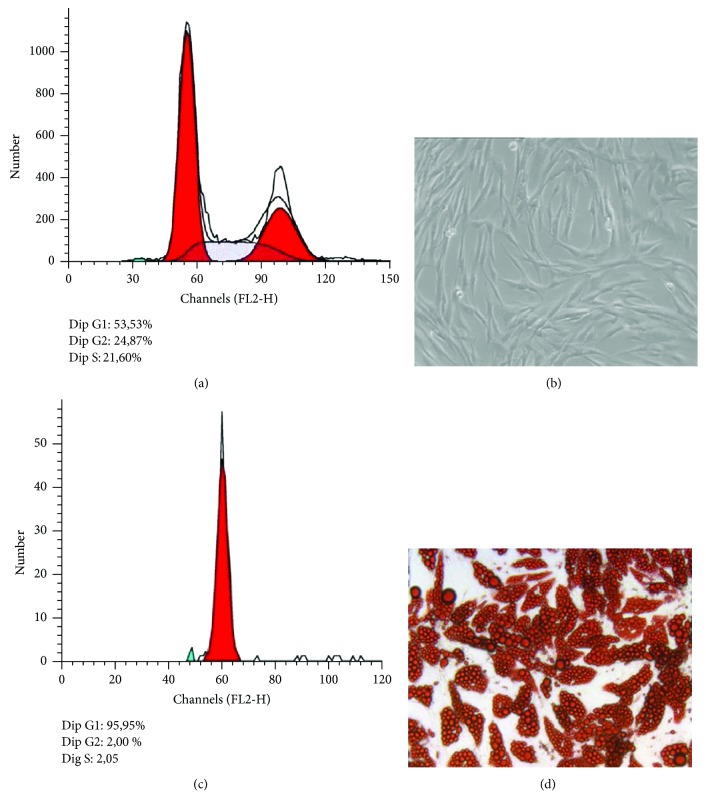
Human preadipocytes SGBS cells: in panel (a) cytometric profile of cell cycle and in panel (b) optical photography. Differentiated human SGBS cells: in panel (c) cytometric profile of cell cycle and in panel (d) optical photography after stained with Oil Red O solution to display the accumulated lipid droplets. The acquired FACS data were analyzed by ModFit LT software.

It is well known that obesity is characterized by adipogenesis and differentiation of preexisting adipocytes; in this study, we have used human preadipocytes (SGBS cells) that are considered physiologically comparable to human adipose tissue in vivo. [23, 24]. These cells are characterized by a high capacity for adipogenic differentiation over many generations and functionally behave like human primary adipocytes. SGBS preadipocytes (Figures 1(a) and 1(b)) were cultured and differentiated into mature adipocytes (Figure 1(c) and 1(d)) as described in the methods section. We examined the effects of adipogenic differentiation on the cell cycle of SGBS preadipocytes induced to differentiate. At the end of the differentiation, the results show arrest of the cell cycle at G1 phase occurred in differentiated cells (Figure 1(c)), whereas cell cycle appears distributed in characteristic G1 S G2 cell cycle phases in nondifferentiated preadipocytes. This result is in line with the characteristic of SGBS preadipocytes that after a complete differentiation arrests growth [25].

### 3.2. Effect of TB on Cell Cycle and Differentiation of Human SGSB Preadipocytes and on U937 Cell Growth

**Figure 2:**
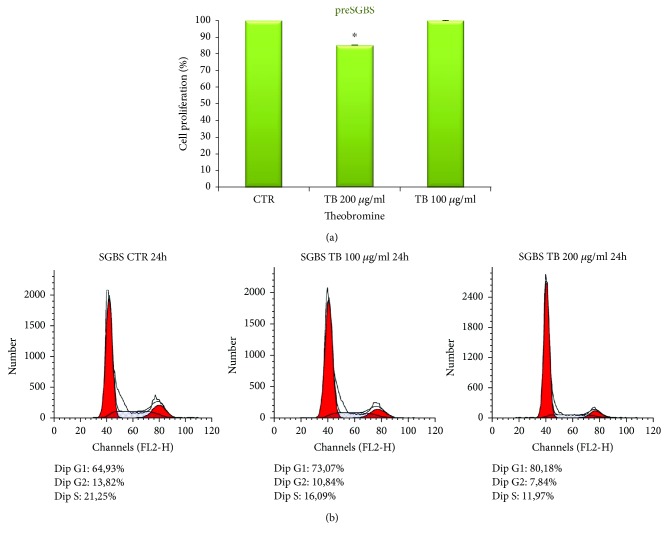
(a) Preadipocytes SGBS were treated with indicated concentration of TB (200 *μ*g/ml and 100 *μ*g/ml) for 48 hrs. Cell proliferation was evaluated by MTT assay. The results are expressed as the percentage of the cell growth inhibition obtained by four experiments (preformed in triplicate). Bar represent the error standard of the percentage mean (^∗^*p* < 0.05). (b) Regulatory effects of TB on cell cycle progression in SGBS preadipocytes cultured in a medium containing 100 and 200 *μ*g/ml TB for 24 h. The cells were stained with a PI solution and analyzed by flow cytometry. The acquired FACS data were analyzed by ModFit LT software.

In order to exclude possible cytotoxic effects of TB, we first performed an MTT assay to examine the influence of TB on SGBS cell viability and proliferation. SGBS cells were treated with different concentrations of TB ranging from 50 to 200 *μ*g/ml for 48 h. The results show that TB produced a limited although statistically significant inhibition of cell proliferation only at the concentration of 200 *μ*g/ml (Figure 2(a)).

The effect of 24 h treatment with TB at the concentration of 100 and 200 *μ*g/ml on cell cycle was investigated. The results illustrated in Figure 2(b) show that TB induces a dose-dependent increase of the percentage of cells in G0/G1 phase and a reduction in S phase. Our results are in agreement with those of Jang et al. [22] that investigated the effects of TB on mouse 3T3-L1 preadipocytes. These authors showed that TB treatment results in arrest of the cell cycle at the G0/G1 phase induced by the regulation of the levels of CDK2, p21, and p27 expression [22].

In order to exclude cytotoxic effects of TB on U937 cells, we performed MTT assays on U937 cell undifferentiated or differentiated with PMA and successively stimulated with LPS to mimic an inflammatory condition. The results suggest that U937 cells unstimulated or stimulated with PMA and LPS were less sensitive than SGBS cells since TB at the concentration of 200 *μ*g/ml did not affect cell growth that was comparable to that of the untreated controls (data not shown).

Taking together, these results point out that TB at the concentrations of 100 *μ*g/ml used in the experiments described below had no cytotoxic effect both in the U937 and SGBS cells.

**Figure 3:**
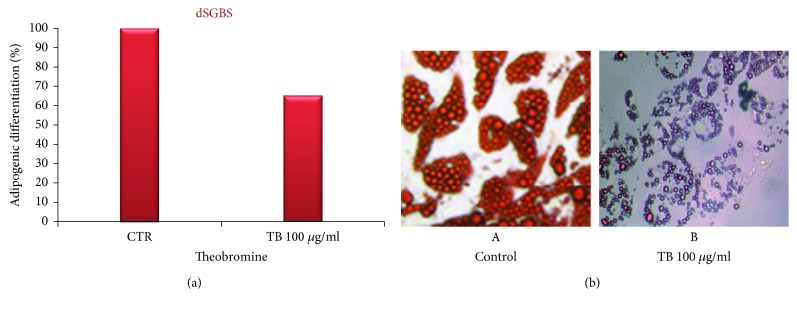
Inhibitory effect of TB on adipogenic differentiation in preadipocytes SGBS. (a) Inhibitory effect of TB on adipogenic differentiation in preadipocytes SGBS. The cells were cultured in a differentiation medium (DM) containing TB (100 *μ*g/ml) added on day 8 from the start of the differentiation process. The cells were stained with Oil Red and were subject to a quantitative analysis of intracellular lipidic accumulation by an ELISA reader (*λ* = 490). The results are expressed as the percentage of the inhibition obtained by four experiments (preformed in triplicate). Bar represent the error standard of the percentage mean (*p* < 0.01). (b) Inhibitory effect of TB on adipogenic differentiation. The SGBS control (A) and the SGBS cultured in a differentiation medium (DM) containing TB (100 *μ*g/ml) added on day 8 from the start of the differentiation process (B). Optical photography.

To investigate whether TB affects adipocyte differentiation, we measured the effect of TB on lipid accumulation during the differentiation of human SGSB preadipocytes. Cells were induced to differentiate to adipocytes with the addition of differentiation medium (MD). TB at the concentration of 100 *μ*g/ml was added to the DM on day 8 and successively every 48 hours until day 14. On day 14, dSGBS cells were stained with Oil Red and photographed (Figure 3(b): left control and right TB treated cells). To quantify staining of fat droplets, Oil Red stain was extracted by adding isopropanol to each well. The aliquots of 100 *μ*l were transferred to 96-well plates and immediately read in an Elisa reader at 490 nm. The results, obtained by the quantitative analysis, confirm the results obtained by microscopy and show a partially but significant inhibition (mean of 35% of inhibition) of adipocyte differentiation following TB treatment with respect to control cells. The results are illustrated in Figure 3(a). Moreover, taking in account that at the concentration of 100 *μ*g/ml the TB treatment is not cytotoxic, it reasonable to affirm that the reduction in the number of differentiation is not linked to a decrease of SGBS cell number provoked by the cytotoxic effects of TB.

The effects of TB on adipocyte differentiation and adipogenesis remains unclear.

Our results are in line with the inhibition of adipogenic differentiation by TB observed by Jang et al. in mouse 3T3-L1 preadipocyte cell line [22]. It is well known that the adipocyte differentiation is associated with multifunctional cellular pathways and requires the sequential regulation of adipogenic and lipogenic genes [26]. In addition, as demonstrated by Jang et al., TB was found to significantly suppress lipid accumulation in a concentration-dependent manner via the decreased expression of PPAR*γ* and C/EBP*α* [22]. Moreover, adenosine acts as an endogenous ligand for adenosine receptors (ARs) in the plasma membrane and is constitutively released from adipose tissues. Adipogenic differentiation results in an increased AR1 expression in mesenchymal stem cells, and activation of this receptor is associated with further increases in adipogenesis [21]. TB exerts antiadipogenic effects through suppression of AR1 signaling [21]. In addition, TB also increased sumoylation of C/EBP*β* since the expression of the small ubiquitin-like modifier (SUMO)-specific protease 2 (SENP2) gene, coding for a desumoylation enzyme, was suppressed by TB [21].

In our experiments, we treated the SGBS cells with TB starting from the eighth day, since at this stage of the adipogenic process, dSGBS show the greatest expression of transcription factor PPAR*γ* and C/EBP*α*, which are the main genes responsible for differentiation and molecular targets [22]. It is reasonable to hypothesize that the same mechanism is involved in the inhibition of human adipocyte differentiation mediated by TB in SGBS cells.

More recently, a xanthene analog [27] has been proposed to control fat cells from adipogenesis. This synthesized xanthene MI-401 was found to have two distinct effects on the regulation of the adipocyte's life cycle. MI-401 efficiently downregulated the expression of transcription factors, PPAR*γ* and C/EBP*α*, and lipogenesis proteins, FAS and FABP4. This downregulation resulted in the inhibition of adipogenesis.

In addition, Mitani et al. [21] showed that in vivo TB administration (0.1 g/kg for 7 days) in ICR mice attenuated gains in the body and epididymal adipose tissue weights in mice and suppressed the expression of adipogenic-associated genes in mouse adipose tissue.

### 3.3. Effect of TB on Cytokines and Chemokines Production by Adipocytes, Macrophages, and Their Cocultures

Inflammation and adipocyte-macrophage interaction has a primary importance in obesity. The interactions between macrophages and adipocytes have influence on cytokine and chemokine production that is involved in inflammatory processes [28]. In order to mimic this inflammatory condition, we investigated the interactions between human-like macrophages and human adipocyte cell lines in vitro in terms of the cytokines and chemokine release in culture supernatants.

**Figure 4:**
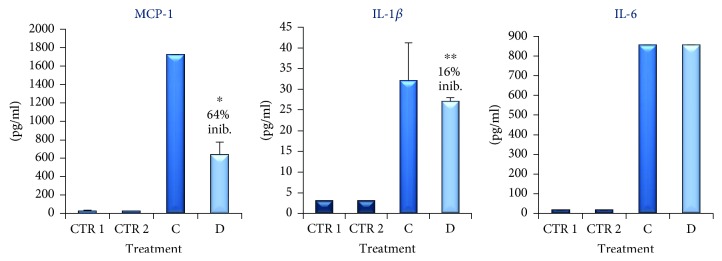
MCP-1, IL-1*β*, and Il-6 accumulation (pg/ml) in the supernatant of coculture of dSGBS and dU937 untreated (column C) or treated for 48 h with TB (100 *μ*g/ml, column D) measured by ELISA. CTR 1 is the supernatant of the SGBS on day 8 of the differentiation process. CTR2 is the supernatant of dU937. The results are expressed as the mean obtained by four experiments (preformed in triplicate). Bar represent the error standard of the mean (^∗^*p* < 0.01, ^∗∗^*p* < 0.05).

Initially, we investigated if proinflammatory molecules such as IL-1*β*, IL-6, and MCP-1, usually involved in obesity, were secreted by adipocytes and macrophages individually. The results obtained showed that in both supernatants of dU937 macrophages (see CTR 1 in Figure 4) and dSGBS after 8 days of culture in MD (see CTR 2 in Figure 4) the levels of IL-1*β*, IL-6, and MCP-1 were extremely low.

In vivo, adipocytes and macrophages are in direct cell-cell interactions, and this is important for the inflammatory reaction [29]. In order to mimic this inflammatory condition, we investigated the interactions between human-like macrophages and human adipocyte cell lines in vitro. To test the effect of this contact, we arranged a coculture of human adipocyte-macrophage in cell-culture plates to allow cell-cell contact. The adipocytes were cocultured with macrophages at the ratio of 10 : 1 (i.e., 10 dSGBS : 1 dU937, respectively) as described in materials and methods. In this condition, the amount of cytokines and chemokines released in the supernatants has been evaluated. As illustrated in Figure 4, we observed a significant increase of IL-6, IL-1*β*, and MCP-1 release in the supernatant of the coculture (see column C in Figure 4). In particular, MCP-1, IL-1*β*, and IL-6 levels were found to be approximately 150-, 10-, and 100-fold higher than controls, respectively. Data are represented as mean ± SEM of three different experiments conducted in triplicate. The higher amounts of these cytokines in the supernatant of dSGBS cocultured with dU937, with respect to the supernatants of the same cell cultured alone, suggest that these cytokines were produced consequently of a direct interaction between preadipocytes and monocytes.

It is well known that, in obesity, the expanding adipose tissue releases a large number of mediators, including TNF-*α*, IL-6, and MCP-1 as well as FFA (free fatty acids), which can interfere with insulin signaling [30]. In addition, (MCP-1)/C-C chemokine receptor 2 (CCR2) pathway also promotes macrophage accumulation into the obese adipose tissue [31]. Our results confirm the involvement of these cytokines and point out that the coculture of dSGBS with dU937 macrophages represents a mechanistically appropriate in vitro translational model of inflamed adipose tissue. Similarly, Chan et al. evaluated the contribution of COX-2/MIF-mediated crosstalk between hypertrophic adipocytes and macrophages to the etiology inflammation in WAT using human SGBS adipocytes and THP-1 macrophages line [32]. Furthermore, Samuvel et al. described a similar human coculture system using U937 monocytes and mature human preadipocytes isolated from human adipose tissue in pericardic fat. In line with our results, the authors described in this model an increase of IL-6 level in the supernatant [33].

IL-1*β*, a major cytokine produced largely by macrophages, is implicated in the development of obesity [34]. On the other hand, the macrophage is the primary source of IL-1*β* that is a key regulator of inflammatory response and that plays a pivotal role in various diseases, including metabolic syndromes and type 2 diabetes [35].

In line with this indication, a marked production of IL-1*β* has been found in the supernatant from macrophage-adipocyte interaction in a “cell to cell” contact (see below).

Many phytochemicals have been found to regulate adipogenesis without any side effects; in particular, TB was previously reported to have effects on the regulation of mice adipocyte differentiation, adipogenic gene expression, signaling pathway, and some cytokine production [21, 22]. However, up to now, the effects of TB on human adipocytes have not been described.

Therefore, we examined the effect of TB on human adipocyte differentiation as described above and on inflammation induced by macrophage-adipocyte interaction.

To determine the effect of TB on the expression of IL-1*β*, IL-6, and MCP-1, we cocultured dSGBS with dU937 in the presence of TB (100 *μ*g/ml). The results, illustrated in Figure 4 (column D), show that TB induces a strong downregulation of MCP-1 whereas reduced moderately but significantly IL-1*β* levels. No significant effect was observed instead on the titer of IL-6 in the supernatant of the coculture.

**Figure 5:**
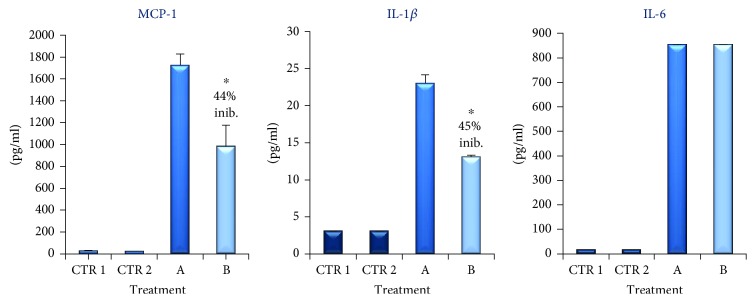
MCP-1, IL-1*β*, and Il-6 accumulation (pg/ml) in the supernatant of dSGBS cells cultured with 100% MacCM (column A) for 48 h alone or in the presence of TB (100 *μ*g/ml, column B) measured by ELISA. CTR 1 is the supernatant of the SGBS on day 8 of the differentiation process. CTR2 is the supernatant of dU937. The results are expressed as the mean obtained by four experiments (preformed in triplicate). Bar represent the error standard of the mean (^∗^*p* < 0.01).

We next examined the cytokine production when dSGBS adipocytes were exposed to MacCM. Therefore, dSGBS cells were cultured with 100% MacCM for 48 h. The data illustrated in Figure 5 (column A) show that inflammatory cytokines IL-6, MCP-1, and IL-1*β* were present in high amounts in the supernatants of preadipocytes cultured with the supernatant of dU937.

This increase is comparable to that obtained following cell to cell contact suggesting that the proinflammatory activation of dSGBS by macrophages probably occurs by soluble mediators. It is important to stress that activation of adipocytes by supernatant of dU937 induces high level MCP-1 that directly triggers the recruitment of macrophages to adipose tissue. The infiltrated macrophages may in turn secrete a variety of chemokines and other cytokines that further promote a local inflammatory response and affect gene expression in adipocytes, resulting in systemic insulin resistance [36].

We following examined whether TB effect could be also detectable in the MacCM-stimulated dSGBS adipocytes. Therefore, dSGBS cells were cultured with MacCM either in the absence or in the presence of TB (100 *μ*g/ml) for 48 h.

When dSGBS adipocytes were incubated with MacCM in the presence of TB, IL-1*β*, and MCP1 levels in culture supernatant were significantly lower than those found in the absence of the drug, whereas the amount of IL-6 was unaffected (see column B in Figure 5). The lack of influence of TB on IL-6 production detected in our experiments is in contrast with the previous results obtained with murine 3T3-L1 cells [22] and with the effect of pentoxifylline and methylxanthine described in human chronic inflammation and visceral obesity [37].

**Figure 6:**
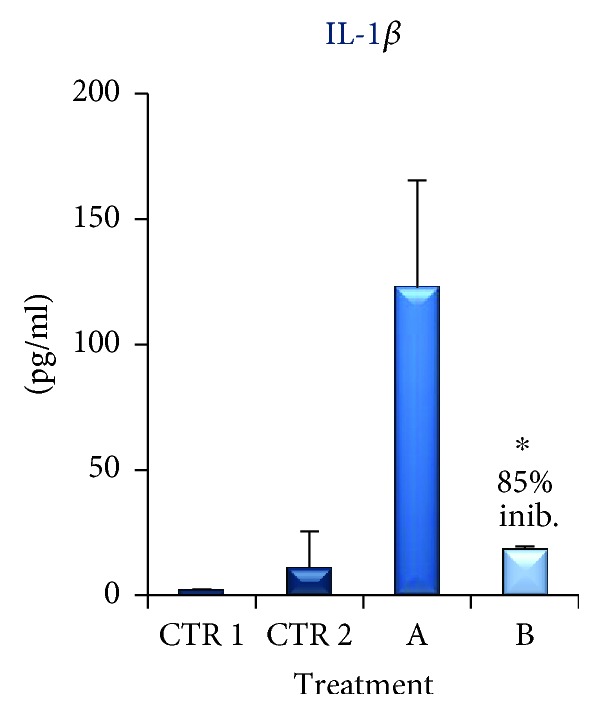
IL-1*β* accumulation (pg/ml) in the supernatant of macrophages dU937 stimulated ApoMC alone (column A) or in combination with TB (100 *μ*g/ml for 48 h, column B) by ELISA. CTR 1 is IL-1*β* accumulation (pg/ml) in the supernatant of the SGBS on day 8 of the differentiation process. CTR2 is IL-1*β* accumulation (pg/ml) in the supernatant of dU937. The results are expressed as the mean obtained by four experiments (preformed in triplicate). Bar represent the error standard of the mean (^∗^*p* < 0.01).

In a third set of experiments, we evaluated the effect of ApoMC alone or in combination with TB (100 *μ*g/ml for 48 h) on IL-1*β* production by macrophages dU937. The results (Figure 6) show that treatment with ApoMC induce a strong upregulation of IL-1*β* by macrophages (see Figure 6, column A) confirming that adipocytes could be able to activate macrophage in adipose microenvironment. The results, illustrated in Figure 6 (column B), show that TB induces a very strong downregulation of IL-1*β* levels.

## 4. Conclusions

Considered together, our data confirm that a crosstalk between human adipocytes and human macrophages occurs in vitro. This interaction is followed by an increase of various inflammatory cytokines and chemokines. Our preliminary results demonstrate that TB is able to reduce, at least in part, the release of proinflammatory molecules. This result reinforces the idea of the use of methylxanthine as modulators of the inflammatory response. TB could be considered as an attracting natural substance potentially able to reduce adipogenesis and inflammation induced by macrophage infiltration in adipose tissue. Apart from the treatment with TB by oral route, a pleasant source of this compound is offered by cocoa and cocoa derivatives. However, dark chocolate contains about 200–300 mg of TB per 40 g chocolate [38], and pharmacokinetics studies in man have shown that plasma peak of the compound reaches the concentration of 7–9 *μ*g/ml after the ingestion of 250 mg of TB [39]. Therefore, the concentrations utilized in our experiments are undoubtedly higher than those that can be reached in vivo after usual doses of ingested chocolate or after oral administration of TB at doses below 1000 mg that are considered acceptable for toxicological point of view [40]. In any case, it is reasonable to hypothesize that chronic assumption of low doses of TB could produce pharmacodynamic effects comparable to those that can be obtained in a short-term and high concentration exposure of target cells.

## Data Availability

Access to this data will be considered by the author upon request.
